# Enhancing the Oral Rehabilitation and Quality of Life of Bisphosphonate-Treated Patients: The Role of Dental Implants

**DOI:** 10.7759/cureus.46654

**Published:** 2023-10-07

**Authors:** Abdulaziz M Altalhi, Albatoul A Alsubaihi, Meshaael M Aldosary, Lama F Alotaibi, Nourah M Aldosariy, Awrad K Alwegaisi, Jalal Y Alghadeer, Abdullah H Aljowayed

**Affiliations:** 1 Dentistry, Ministry of Health, Riyadh, SAU; 2 Dentistry, King Saud Bin Abdulaziz University for Health Sciences, Riyadh, SAU; 3 Dentistry, King Khalid Hospital, Al-Kharj, SAU; 4 Dentistry, Qassim University, Buraydah, SAU; 5 Dentistry, King Saud Medical City, Riyadh, SAU

**Keywords:** periodontics, dental implant survival, osteointgeration, bronj, dental implant

## Abstract

The purpose of this review is to examine the literature on the topic of bisphosphonate-related osteonecrosis of the jaw (BRONJ) and dental implant failure in patients undergoing bisphosphonate (BP) therapy who also received dental implants before, during, or after BP treatment, as compared to healthy patients. This research followed the guidelines in the Preferred Reporting Items for Systematic Reviews and Meta-analysis (PRISMA) statement. The "PICO" or population, intervention, comparison, and outcome clinical question was as follows: does the insertion of dental implants in patients receiving bisphosphonate therapy increase the failure and loss of implants or the incidence of bisphosphonate-related osteonecrosis of the jaw compared to healthy patients? The articles published in PubMed/Medical Literature Analysis and Retrieval System Online (MEDLINE) up to July 1, 2023, were retrieved using a mix of Medical Subject Heading (MeSH) words and their entry terms. The absence of randomized clinical trials examining this issue underscores the need for additional studies with extended follow-ups to answer outstanding questions. Because of the potential for BRONJ and implant failure, patients receiving bisphosphonate medication should exercise caution when planning dental implant surgery. In addition, when such procedures are carried out, the patient's entire systemic condition must be considered.

## Introduction and background

Dental implants have gained increasing popularity as a reliable and aesthetically appealing solution for tooth replacement [1]. However, the use of bisphosphonates (BP), which are commonly prescribed for conditions such as osteoporosis, cancer-related conditions, and metabolic bone diseases, can potentially compromise the efficacy of dental implant treatment [2].

Bisphosphonates, including zoledronic acid, have emerged as vital pharmacological agents in the management of various bone disorders. Their mechanisms of action involve inhibiting osteoclast-mediated bone resorption by binding to the bone surface and disrupting vital cellular processes essential for osteoclast function [3,4]. Moreover, certain bisphosphonates, such as zoledronic acid, exhibit additional antiangiogenic properties, hindering the formation of new blood vessels [5]. This multifaceted pharmacological profile contributes not only to the preservation of bone density and reduction in fracture susceptibility but also to potential repercussions on dental implant outcomes.

Successful osseointegration is crucial for the long-term success of dental implants, and bisphosphonates may impact this process. Patients using bisphosphonates are at an increased risk of developing bisphosphonate-related osteonecrosis of the jaw (BRONJ), a condition characterized by necrotic bone in the maxillofacial region that fails to heal within the expected timeframe [6]. It is important for patients and healthcare providers to be aware of this risk and take necessary precautions during dental implant procedures.

In light of these considerations, this comprehensive review aims to explore the pharmacology of bisphosphonates, including their mechanisms of action and effects on bone metabolism. By providing insights into the intricate interplay between bisphosphonates and dental implants, this review aims to shed light on the potential impact of bisphosphonates on osseointegration and dental implant outcomes. Furthermore, controversies and challenges in this field will be addressed to provide a consolidated understanding of the current knowledge.

## Review

Research methodology

To conduct our research, we utilized PubMed, Google Scholar, Embase, and Medical Literature Analysis and Retrieval System Online (MEDLINE) databases, employing the keywords "Bisphosphonates, BRONJ, Osseous surgery+BRONJ, Open flap debridement+BRONJ, scaling and root planning+BRONJ." We included all clinical trials and systematic reviews that matched our search criteria. The articles that were deemed inappropriate based on the title alone were excluded. Additionally, a thorough evaluation of the abstracts helped us select the most relevant articles pertaining to our topic.

Bisphosphonates and dental clinic

The most significant adverse effect of bisphosphonates is the development of bisphosphonate-related osteonecrosis of the jaw (BRONJ), which has a significant impact on various dentoalveolar procedures. BRONJ refers to the necrotic bone in the maxillofacial region that does not heal within six to eight weeks. It is unrelated to radiation exposure or other external factors [7]. BRONJ is associated with poor oral hygiene and preexisting inflammatory conditions such as periapical pathology and periodontal diseases. Therefore, patient education is critical in preventing BRONJ and optimizing oral health. This approach should be undertaken before and during antiresorptive (AR) therapy and involve proactive dental care, fluoride application, and chlorhexidine rinses. To preserve dental function, preventive measures such as caries control, minimally invasive restorative dentistry, and nonsurgical endodontic therapy are pivotal. Additionally, the teeth that cannot be saved or have a poor prognosis should be extracted before initiating antiresorptive therapy. Moreover, enhancing overall patient health is invariably recommended, encompassing actions such as smoking cessation and diabetes optimization [8].

The precise mechanisms by which bisphosphonates disrupt the normal healing process in the jawbone, resulting in BRONJ, are not fully understood. It is presumed that bisphosphonates inhibit osteoclast activity, resulting in diminished bone resorption and remodeling. This impaired resorption of injured bone tissue can disrupt the natural healing process. Furthermore, specific bisphosphonates such as zoledronic acid have been found to impede the formation of new blood vessels at the injury site, consequently delaying the healing process [9].

In the maxillofacial region, the bone is subjected to various stresses and forces during activities such as mastication, swallowing, and speaking. In a healthy individual, the body employs numerous mechanisms, including osteoclast activity, to facilitate the healing process. Notably, the alveolar region of the mandible, which houses the dental sockets, is particularly susceptible to BRONJ. This vulnerability arises due to the considerable stress and strain endured during mastication, coupled with the significantly higher bone turnover in this region compared to other parts of the body (approximately 10 times higher than in the tibia) [8]. Invasive dental procedures, such as tooth extraction, can disrupt the delicate mucosal and periosteal barrier between the teeth and bones. This disruption increases the susceptibility to the development of BRONJ [10].

Bisphosphonates function by inhibiting osteoclasts, the cells responsible for the breakdown of injured bone cells, thereby impeding the healing process. Consequently, patients undergoing bisphosphonate therapy may encounter challenges in healing their jawbone after a tooth extraction, rendering them more predisposed to BRONJ. The current optimal estimation for the risk of BRONJ following extraction is approximately 0.5% for patients exposed to oral bisphosphonates [11], while intravenous bisphosphonates elevate this risk to about 14.8% [12]. Moreover, this risk is directly proportional to the duration of medication use and the patient's age [13]. To reduce the development of BRONJ, it is recommended to employ primary closure techniques during dental procedures and administer postoperative antibiotics. These measures can help promote proper healing and may mitigate the development of BRONJ [14].

Implant survival rate

The dental implant success rate serves as a crucial benchmark in evaluating the efficacy of dental implantology within the context of bisphosphonate-treated patients. This metric varies depending on the medication type and the mode of administration. Several studies have explored bisphosphonates' impact on bone health and the outcomes of dental implant procedures in such patients. For instance, Gelazius et al. (2018) conducted an extensive systematic review, revealing significant findings. According to their findings, patients who received bisphosphonate therapy directly into their mouth had a significantly higher success rate for implants at 98.8% compared to those who received the treatment intravenously, achieving a slightly lower rate of 91%. These findings underscored the potential for achieving favorable dental implant results in bisphosphonate-treated patients, offering reassurance to both clinicians and patients [15]. Another recent systematic review and meta-analysis by Sulaiman et al. (2023) showed that implants in bisphosphonate-treated patients had a higher failure risk than non-bisphosphonate-treated patients [16]. However, Papadakis et al. (2021) [17] introduced a critical caveat to this discussion. They noted the challenge in making definitive claims about the impact of antiresorptive medication on dental implant success rates. This challenge arises due to the limitations inherent in many studies within this domain, such as small sample sizes, the absence of control groups, and relatively short follow-up periods. Consequently, it becomes difficult to establish the precise success rate of dental implants in patients undergoing antiresorptive medication. Consequently, the accurate assessment of the risk associated with medication-related osteonecrosis of the jaw (BRONJ) development in dental implant patients remains elusive.

Implant protocol

Achieving successful and safe dental implant placement in patients undergoing bisphosphonate treatment requires the meticulous implementation of specific implant protocols. In their review, Sher et al. (2021) [18] examined the literature to determine the best protocol for these patients. Their study emphasized the need for a comprehensive patient evaluation, including a detailed medical history review and a careful assessment of BRONJ risk factors. Furthermore, the authors stressed adopting conservative surgical techniques and advocating for a cautious and measured approach during the surgical procedure itself. They also highlighted the significance of vigilant postoperative monitoring and strict adherence to carefully designed aftercare regimens to mitigate potential complications. These findings emphasize the crucial role of tailored implant protocols in securing successful outcomes for patients affected by BRONJ.

Drug holiday

Drug holiday involves temporarily discontinuing bisphosphonate treatment before and after dental implant surgery to mitigate the risk of BRONJ. Ruggiero et al. [19] investigated the American Association of Oral and Maxillofacial Surgeons' (AAOMS) stance on BRONJ. They emphasized the crucial need for close collaboration between oral surgeons and the physicians prescribing bisphosphonates. Their perspective advocates for an individualized approach to drug holidays, taking into account the patient's medical history, type of medication, and duration of bisphosphonate therapy. A well-considered drug holiday can significantly enhance the safety of dental implant procedures for individuals in this category. For patients undergoing bisphosphonate therapy, it is important to find a balance between the risks of BRONJ and the benefits of oral rehabilitation when implementing a drug holiday.

However, the utilization of antiresorptive drug holidays in patients undergoing dentoalveolar surgery to mitigate the risk of BRONJ has been controversial in 2014, at the time of the previous AAOMS position paper, and in 2021. Despite the acceptance and recommendation of drug holidays by various international professional societies, the evidence supporting or refuting such an approach remains inconclusive. The challenge in establishing the efficacy of drug holidays arises from the infrequency of BRONJ in these patient populations, resulting in a lack of reported cases and insufficient data from randomized controlled trials to establish effective treatment protocols [19].

Papadakis et al. (2021) [17] evaluated the success and safety of dental implantology within this patient cohort. They found that carefully timing and managing drug holidays can lower the risk of BRONJ development and still allow for successful dental implant placement. This approach allows patients to experience improved oral function and an enhanced quality of life, all without compromising their overall health.

However, a recent meta-analysis conducted by Aboalela et al. (2022) [20] analyzed eight articles with 6808 subjects and concluded that there was no significant difference in the development of BRONJ following a tooth extraction procedure between the drug holiday group and the non-drug holiday group. The study concluded that drug holidays with antiresorptive (AR) medications will not minimize the risk of BRONJ and cannot be advised based on the available evidence. Thus, the authors suggested that more definitive conclusions can be drawn from large prospective studies and good-quality randomized trials.

Main complications

Bisphosphonates Affect Implant

A systematic review by Granate-Marques et al. [21] identified several studies reporting an increased risk of bisphosphonate-related osteonecrosis of the jaw (BRONJ) associated with dental implants placed in the posterior jaw of patients on long-term bisphosphonate therapy, particularly in those concurrently receiving systemic corticosteroids. While dental implantology has generally exhibited positive outcomes in patients receiving bisphosphonate treatment, it is important to consider various potential complications, including an elevated risk of BRONJ, implant failure, and peri-implantitis. Bisphosphonates disrupt normal bone turnover and reduce the resistance of peri-implant bone to oral bacteria, thereby contributing to these risks [21]. Consequently, caution should be exercised when contemplating implant procedures for individuals on long-term bisphosphonate therapy [22]. These patients ought to undergo careful evaluation to assess their risk of BRONJ and other complications and be placed on a regular long-term recall schedule for close monitoring and management [22]. Therefore, careful consideration is essential when considering implant treatment.

Implant in Relation to BRONJ

A report of implant-related necrosis can be divided into early (implant surgery-triggered) or late (implant presence-triggered) category. The majority of implant-related necrosis cases were not related to the initial implant surgery but occurred late, often at sites where implants were placed prior to the initiation of bisphosphonate therapy. These cases are typically presented as en bloc failure, where the osseointegration of the implants is maintained within the sequestrum, and this may be related to macro-cracks around the dental implant [23,24].

Clinical presentation of BRONJ

The staging system for BRONJ was first introduced in the 2009 AAOMS position paper. It was later improved in the 2014 position paper to give a more accurate description of all aspects of BRONJ's clinical presentation [19]. These classifications of BRONJ include the following stages.

Stage 0

Patients are usually asymptomatic. This stage involves presenting nonspecific symptoms, including toothache, dull and aching pain in the jaw radiating to the temporomandibular joint, changes in neurosensory function, and pain in the sinus, mainly the maxillary sinus. In clinical findings, there may be loosening of the teeth and swelling intraorally or extraorally. However, these findings are not associated with any periodontal disease. Radiographic findings may include the loss or resorption of the alveolar bone, no new bone in the extraction socket, and the concealment of the periodontal ligament.

Stage 1

This is also an asymptomatic stage. In this stage, there may be exposed bone, or the necrotic bone may also be exposed, which is not associated with any infection or inflammation. The radiographic finding may be the loss or resorption of alveolar bone.

Stage 2

During this symptomatic stage, there may be signs of infection, inflammation, necrosis, exposed bone, and fistulas that can extend into the bone.

Stage 3

This stage includes stage 2 findings, as well as any instances of exposed bone outside of the alveolar area. There is a risk of pathological fractures or the formation of an external fistula. Additionally, bone breakdown may occur beyond the inferior borders of the mandible.

Pharmacology of bisphosphonates

After more than 50 years of research on bisphosphonates, there has been significant progress in understanding their pharmacology. These compounds play a crucial role in preventing excessive bone resorption caused by osteoclasts, thus helping to maintain bone mass and structure in conditions such as postmenopausal osteoporosis and metastatic bone diseases [25]. Bisphosphonates can be classified into two generations based on their mechanism of action and discovery date: non-nitrogen-containing bisphosphonates (first generation) and nitrogen-containing bisphosphonates (second and third generations). The examples of first-generation bisphosphonates include etidronate, clodronate, and tiludronate, while second-generation bisphosphonates include pamidronate, alendronate, and ibandronate. The third generation includes zoledronate and risedronate [26].

The positioning and distance of the nitrogen moiety in relation to the phosphonate groups significantly influence the antiresorptive potency of nitrogen-containing bisphosphonates [27,28]. Therefore, understanding the pharmacokinetic and pharmacodynamic aspects of bisphosphonates is vital for comprehending their overall pharmacology.

Pharmacokinetic

Bisphosphonates, including alendronate, risedronate, and ibandronate, are commonly administered orally on a weekly or monthly basis. In particular, alendronate and risedronate are given weekly, while risedronate and ibandronate are taken monthly. Intravenous administration is reserved for bisphosphonates such as zoledronic acid, pamidronate, and ibandronate. However, non-nitrogen-containing bisphosphonates, which are no longer widely used due to their low effectiveness and significant adverse effects, are typically administered orally [29,30].

When taken orally, bisphosphonates are absorbed in the gastrointestinal tract through a combination of paracellular and active transport mechanisms [31]. However, research has shown that the absorption of bisphosphonates can be harmful to epithelial layers [32]. Additionally, oral bisphosphonates generally have low bioavailability, with approximately 99% of the orally administered medication being excreted unchanged in feces. However, certain types of bisphosphonates can achieve 100% absorption when taken with calcium- or magnesium-rich foods, drinks, or medications [33]. Conversely, medications that increase the stomach's pH, such as ranitidine and omeprazole, can enhance the effectiveness of bisphosphonates [33]. Caproic acid and ethylenediaminetetraacetic acid (EDTA) have also shown potential in improving the oral bioavailability of bisphosphonates.

The protein binding of bisphosphonates can vary widely (5%-90%), depending on factors such as the specific bisphosphonate, concentration, pH, calcium, and studied species [34]. However, it is still unclear whether protein binding affects renal excretion, delivery to bone tissue, and resorption from bone tissue.

Bisphosphonates selectively target the bone rather than soft tissues due to their high affinity for exposed surfaces of hydroxyapatite bone mineral. They distribute unevenly within the skeletal system, with higher concentrations found in sites with high bone turnover, particularly trabecular bone, rather than cortical bone [35-37]. Bisphosphonates do not undergo first-pass metabolism and phase I or phase II metabolism. However, non-nitrogen-containing bisphosphonates, such as etidronate, clodronate, and tiludronate, transform into cytotoxic adenosine triphosphate (ATP) analogues intracellularly, which is necessary for these bisphosphonates to inhibit osteoclast-mediated bone resorption [38]. After release, bisphosphonates may be reabsorbed onto bone surfaces, leading to their detection in urine for years, even after treatment cessation [39,40].

For the treatment of osteoporosis in both males and females, alendronate is usually given in a weekly dose of 70 mg. In postmenopausal females, a lower dose of 35 mg once weekly can be used for osteoporosis prevention. Alendronate is also taken orally to treat Paget's disease of the bone, with a dose of 40 mg once daily for six months [41,42]. Risedronate is administered orally in a dose of 30 mg daily for two months or 35 mg once weekly or 150 mg once a month. Ibandronate sodium can be given orally in a dose of 150 mg once a month or intravenously in a dose of 3 mg once a month. Zoledronic acid is administered intravenously at a dose of 4 mg to 5 mg over at least 15-30 minutes every 12 months for osteoporosis management. Pamidronate is administered intravenously slowly in a dose of 30 mg to 60 mg every three to six months for the management of hypercalcemia of malignancy, Paget's disease, and bone metastasis [43-45]. It is advised that patients with inadequate dietary intake supplement with 1000-1200 mg/day of calcium and 800-1000 international units/day of vitamin D [42]. To ensure proper absorption and reduce the risk of side effects, patients should take the medication orally in the morning on an empty stomach, at least 30 minutes before eating, drinking (except water), or taking other medications. Additionally, they should remain upright for at least 30 minutes after taking the medication to prevent esophageal ulceration or irritation.

Importantly, bisphosphonates have potential side effects that need to be considered. The common adverse reactions can include gastrointestinal symptoms such as esophageal irritation, dysphagia, and abdominal pain. While rare, osteonecrosis of the jaw (BRONJ) and atypical femoral fractures have been reported as serious side effects. Patients should be educated about these risks and advised to seek medical attention if they experience any unusual symptoms or concerns during bisphosphonate treatment.

In summary, bisphosphonates, both nitrogen-containing and non-nitrogen-containing, inhibit bone resorption by impairing osteoclast activity. Nitrogen-containing bisphosphonates, such as alendronate, risedronate, ibandronate, and zoledronic acid, play a significant role in reducing fracture risk in osteoporosis and have shown promising anti-tumor activity. The choice of administration route, whether oral or intravenous, depends on the specific bisphosphonate and the patient's circumstances. Following the prescribed medication regimen, including fasting and maintaining an upright posture, is crucial for proper absorption and minimizing side effects. Regular monitoring and communication with healthcare professionals are essential for the optimal management of bisphosphonate therapy.

Pharmacodynamic

Bisphosphonates, regardless of whether they are nitrogen-containing or non-nitrogen-containing, work by inhibiting bone resorption through the impairment of osteoclast activity. Non-nitrogen-containing bisphosphonates, which are no longer used in practical situations, are metabolized intracellularly to produce nonfunctional molecules that compete with ATP in the cell's energy metabolism. This ultimately leads to osteoclast apoptosis and the prevention of bone breakdown [46]. However, non-nitrogen-containing bisphosphonates have been associated with the inhibition of bone mineralization and the potential to cause osteomalacia [42].

On the other hand, nitrogen-containing bisphosphonates, such as alendronate, risedronate, ibandronate, and zoledronate, inhibit the farnesyl pyrophosphate synthase (FPPS) enzyme in the mevalonate pathway. This inhibition results in the reduced prenylation of signal transduction proteins involved in osteoclast-mediated bone resorption, such as Rac, Ras, and Rho [47,48]. Additionally, bisphosphonates help to preserve the viability of osteoblasts and osteocytes [49]. As a result, the main clinical application of bisphosphonates is reducing fracture risk in osteoporosis. Moreover, bisphosphonates have shown anti-tumor activity in preclinical trials, inhibiting cell migration, invasion, and metastasis [50,51]. For example, zoledronate affects signaling pathways such as microRNA-21 (miR-21)/phosphatase and tensin homolog (PTEN)/protein kinase B (Akt) and activin pathways [52].

Bone turnover markers

Performing dental procedures on patients who are taking bisphosphonates may heighten the likelihood of BRONJ. This increased risk is likely due to the elevated demand for bone turnover triggered by invasive dental procedures, which leads to a greater number of binding sites available for bisphosphonates. As a result, bisphosphonates tend to accumulate locally in the jaw [53]. Therefore, it is hypothesized that evaluating bone turnover markers could play a crucial role in promptly detecting BRONJ and enabling timely intervention.

Bone turnover consists of two stages: resorption, which involves removing old bone, and formation, which involves depositing new bone. Evaluating bone turnover usually involves measuring specific biochemical markers in serum and urine. The markers of bone resorption include N-terminal telopeptide (NTX) and C-terminal telopeptide (CTX), which are breakdown products of collagen [35]. The markers of bone formation include procollagen type I carboxy-terminal propeptide (P1CP) and procollagen type I N-terminal propeptide (P1NP), which are propeptides of type I collagen [54]. The levels of these markers can vary due to changes in systemic and local bone turnover. Systemic alterations in bone turnover can occur in postmenopausal and glucocorticosteroid-induced osteoporosis, while local changes can be seen in conditions such as osteoporotic fractures, Paget's disease of the bone, and bone metastases from solid tumors [55]. Furthermore, the levels of bone turnover markers can vary based on factors such as the specific metabolic bone disease, the marker being measured, the measurement method, the use of bisphosphonates, the route of administration, and the dosage regimen [55].

The route of administration for bisphosphonates can impact the levels of bone turnover markers in patients. Research has demonstrated that individuals receiving intravenous bisphosphonates, such as zoledronic acid (4 mg or 5 mg), ibandronate (3 mg), and pamidronate (75 mg), for postmenopausal osteoporosis display different levels of bone turnover markers compared to those treated with oral bisphosphonates, such as alendronate (70 mg/week) or risedronate (35 mg/week) [35]. Specifically, it was observed that the intravenous administration route led to a rapid decline in bone resorption markers, reaching the lowest levels within a few days. In contrast, oral treatment required a longer time span, with the lowest levels reported after approximately three months [35].

Current evidence does not provide a conclusive assessment of the clinical effectiveness of bone turnover markers in determining the risk of BRONJ. A systematic review conducted recently found that these markers are not reliable predictors for predicting BRONJ risk. The study recommends further investigation into the potential of angiogenesis and endocrine markers for accurately assessing BRONJ risk [56].

## Conclusions

In conclusion, dental implant procedures in bisphosphonate-treated patients require careful consideration and management due to the potential complications associated with bisphosphonate use. The success rate of dental implants in these patients varies depending on factors such as the medication type and mode of administration. While some studies have reported high success rates for implants in patients receiving bisphosphonate therapy, there is also an elevated risk of medication-related osteonecrosis of the jaw (BRONJ) and implant failure. Implementing specific implant protocols, conducting thorough patient evaluations, and practicing conservative surgical techniques can help mitigate the risks of BRONJ and implant failure. The timing and management of drug holidays, where bisphosphonate treatment is temporarily discontinued before and after dental implant surgery, remain a topic of debate, and more research is needed to establish its effectiveness in reducing the risk of BRONJ.

Monitoring bone turnover markers may play a role in detecting BRONJ early and enabling timely intervention, although their clinical effectiveness in predicting BRONJ risk is still uncertain. Overall, close collaboration between oral surgeons and prescribing physicians, individualized treatment planning, regular post-implant maintenance, and vigilant monitoring are essential for achieving successful outcomes in bisphosphonate-treated patients undergoing dental implant procedures. However, it is important to acknowledge the limitations of the current evidence in this area, including the scarcity of large-scale prospective studies and randomized controlled trials. Further research is needed to strengthen our understanding of the optimal management strategies and to develop more reliable predictors of BRONJ risk in patients on bisphosphonate therapy. By continuously improving our knowledge and refining treatment protocols, we can provide safer and more effective dental implant procedures for bisphosphonate-treated patients.
